# Ascending Vaginal Infection in Mice Induces Preterm Birth and Neonatal Morbidity

**DOI:** 10.1016/j.ajpath.2025.01.008

**Published:** 2025-01-30

**Authors:** Ashley K. Boyle, Konstantina Tetorou, Natalie Suff, Laura Beecroft, Margherita Mazzaschi, Rajvinder Karda, Mariya Hristova, Simon N. Waddington, Donald Peebles

**Affiliations:** ∗ Elizabeth Garrett Anderson Institute for Women's Health, University College London, London, United Kingdom; † Department of Women and Children's Health, St Thomas' Hospital, King's College London, London, United Kingdom; ‡ Faculty of Health Sciences, Wits/South African Medical Research Council Antiviral Gene Therapy Research Unit, Johannesburg, South Africa

## Abstract

Preterm birth (PTB; delivery before 37 weeks), the main cause of neonatal death worldwide, can lead to adverse neurodevelopmental outcomes, as well as lung and gut pathology. PTB can be associated with ascending vaginal infection. Ascending *Escherichia coli* infection in pregnant mice induces PTB and reduces pup survival. The current study demonstrated that this model recapitulates the pathology observed in human preterm neonates (namely, neuroinflammation, lung injury, and gut inflammation). In neonatal brains, there is widespread cell death, microglial activation, astrogliosis, and reduced neuronal density. The utility of this model was validated by assessing the efficacy of maternal cervical gene therapy with an adeno-associated viral vector containing human β defensin 3. This improved pup survival and reduced tumor necrosis factor alpha mRNA expression in perinatal pup brains exposed to *E. coli*. This model provides a unique opportunity to evaluate the therapeutic benefit of preterm labor interventions on perinatal pathology.

Preterm birth (PTB; delivery before 37 weeks) is the leading cause of neonatal morbidity and mortality worldwide.^1^^,^^2^ This is a serious global health problem, affecting approximately 11% of all pregnancies and 15 million babies a year.^3^, ^4^, ^5^ Risk factors include extremes in maternal age, body mass index, socioeconomic disadvantage, smoking, multiple pregnancies, and a previous PTB.^6^^,^^7^

Despite extensive research, treatment strategies have proved largely ineffective. This is likely because of the multifactorial nature of spontaneous PTB. The cervix, a physical and immunologic barrier containing immune cells and producing antimicrobial peptides, plays a key role in preventing bacterial invasion. However, at least 40% of spontaneous PTBs are associated with microbes, which are thought to ascend from the vagina into the uterus, via the cervix.^8^ The microorganisms commonly linked with PTB have relatively low pathogenicity, and cases of chorioamnionitis are mostly polymicrobial. Species of *Mycoplasma*, *Ureaplasma*, *Fusobacterium*, and *Streptococcus* have been found in the amniotic fluid in those most at risk.^9^^,^^10^ Vaginal *Lactobacillus iners* dominance has been linked to PTB risk in a UK population, whereas *Lactobacillus crispatus* dominance has been associated with term delivery.^11^, ^12^, ^13^

The fetal immune system is primed *in utero* through exposure of the placenta, lungs, gut, and skin to microbes, as well as the vertical transmission of maternal immune cells.^14^^,^^15^ When delivered, babies exposed to infection/inflammation have an increased risk of periventricular leukomalacia, intraventricular hemorrhage, and early-onset neonatal sepsis. These conditions increase the likelihood of long-term complications resulting in neurodevelopmental disorders, such as cerebral palsy, and respiratory-related conditions, such as bronchopulmonary dysplasia, and necrotizing enterocolitis (NEC).^16^^,^^17^

PTB can be induced in mice by ascending vaginal infection with a pathogenic *Escherichia coli* strain associated with human neonatal meningitis. The proportion of pups born alive is reduced and, in surviving pups, there is evidence of neuroinflammation.^18^ This model was used to investigate a novel therapeutic, demonstrating that adeno-associated viral (AAV) vector gene therapy to increase levels of the antimicrobial peptide human β defensin 3 (HBD3) in the cervix can reduce ascending infection and improve pup survival.^19^

Here, the fetal and neonatal outcomes following exposure to ascending vaginal infection were characterized, assessing the fetal lungs and gut, as well as further interrogating fetal and neonatal neuropathology. The impact of pre-treating dams with cervical HBD3 gene therapy on perinatal neuroinflammation was assessed.

## Materials and Methods

### AAV Vector Production

An AAV8 bicistronic vector was produced, encapsidating a single-stranded DNA sequence containing the HBD3 gene followed by the engineered green fluorescent protein (eGFP) gene, each under the transcriptional activity of a separate cytomegalovirus (CMV) promotor. An AAV8 vector containing CMV-eGFP was used as a vehicle control. For NF-κB biosensor experiments, an AAV8 serotype vector containing a minimal promoter upstream of the NF-κB response element, followed by a firefly luciferase and 2A-eGFP transgene, was developed.^20^

AAV production and purification followed standard procedures. Briefly, HEK293T cells were transfected with pAAV-CMV-hDEFB103B-CMV-eGFP or pAAV-CMV-eGFP (Vector Biolabs, Malvern, PA), using polyethylenimine (PEImax; Polysciences Inc., Warrington, PA). Cells were incubated at 37°C for 72 hours, then collected, centrifuged, and freeze thawed in lysis buffer. Both cell supernatant and cell lysate were benzonase treated, then clarified via centrifugation and 0.22-μm filtration before purification. AAVs were purified by high-performance liquid chromatography (ÄKTAprime plus; GE Healthcare, Buckinghamshire, UK) with a POROS CaptureSelect AAV Resin (Thermo Fisher Scientific, Oxford, UK) column. Purified vector fractions were dialyzed against phosphate-buffered saline (PBS) overnight (Slide-A-lyzer dialysis cassette 10,000 MWCO; Thermo Fisher Scientific). Viral genome titers were quantified by quantitative PCR (qPCR).

### Mouse Model of Ascending Vaginal Infection

All animal studies were conducted under a UK Home Office Licence (PAD4E6357) in line with the Animal Scientific Procedures Act (1986) and following the Animal Research: Reporting of In Vivo Experiments (ARRIVE) guidelines 2.0.^21^ Temperature (19°C to 23°C) and humidity (approximately 55%) were tightly controlled at all times, with constant 12-hour light/dark cycles. Virgin female C57BL/6 Tyr^c−2J^ mice (Charles River, Kent, UK), aged 6 to 12 weeks, were time mated [embryonic day (E) 0.5, designated when vaginal plug was found].

On E16.5, mice were anesthetized by the inhalation of isoflurane (5% for induction, 1.5% for maintenance in oxygen). *E. coli* K1 A192PP-*lux2*, modified to contain the lux operon from *Photorhabdus luminescens*,^22^ was delivered intravaginally to induce preterm delivery, as previously described.^18^ Briefly, 20 μL of midlogarithmic-phase *E. coli* (1 × 10^2−3^ colony-forming units resuspended in PBS) or PBS (vehicle control) was delivered into the vagina of pregnant mice using a 200-μL pipette tip, followed by 20 μL of 20% Pluronic F-127 gel (Sigma-Aldrich, Dorset, UK) to prevent leakage. Animals were allowed to recover from anesthesia, then caged individually and monitored by closed-circuit television for signs of labor and the delivery of pups. Time to delivery was recorded as the number of hours from the time of intravaginal administration of *E. coli* or PBS to the delivery of the first pup. The number of live/dead pups was recorded within 24 hours of their delivery and a percentage calculated per litter.

In a separate cohort, on the day of birth, AAV8 containing a firefly luciferase reporter driven by the NF-κB response elements was administered intracranially to neonatal mice exposed to *E. coli* or PBS *in utero*. Neonatal mice were imaged daily up to 21 days old to assess NF-κB response in the brain.

In a third cohort, pregnant C57BL/6 Tyr^c−2J^ females (aged 6 to 12 weeks) were anesthetized on E13.5, and 10 μL of AAV8-HBD3 or AAV8-GFP was delivered intravaginally using a sterile 200-μL pipette tip (1 × 10^12^ genomic copies/mL diluted in PBS), followed by 10 μL of AK012 thermosensitive gel (PolySciTech, West Lafayette, IN). Administration of *E. coli* or PBS on E16.5 was then performed as already described.

### Bioluminescent Imaging

Mice were imaged for 2 minutes using a cooled charged-coupled device camera (IVIS Lumina; Perkin Elmer, Coventry, UK). Dams were anesthetized with isoflurane as above.

### Tissue Collection and Processing

Perinatal pups were sacrificed by decapitation on E18.5 (48 hours after intravaginal infection) to harvest brain, lungs, and gut (one to three pups per litter collected from the right horn). Dams and postnatal day (P) 7 and P14 pups were anesthetized using isoflurane, the right atrium was incised, and PBS was injected into the left ventricle. P7 brains were cut into three coronal sections: the forebrain, the mid brain region, and the hindbrain/cerebellum. Tissues for qPCR and enzyme-linked immunosorbent assay analyses were snap frozen and stored at –80°C until required. For immunohistochemistry, brain and lung samples were fixed in 4% paraformaldehyde for 24 to 48 hours, then stored in 30% sucrose at 4°C. Tissues were frozen on dry ice and a cryostat (LEICA CM1900; Leica, Newcastle upon Tyne, UK) was used at –20°C to cut 50 serial sections (40 μm thick) of the tissues, beginning from the fusion of the corpus callosum of the brain. Frozen sections were stored at –80°C.

### RNA Extraction, cDNA Synthesis, and qPCR

Total RNA was extracted from E18.5 brains, lungs, and gut and P7 forebrain and mid brain samples by RNAeasy mini kit (Qiagen, Germantown, MD), according to the manufacturer's instructions, and quantified on a FLUOstar Omega Microplate Reader (BMG Labtech Ltd, Aylesbury, UK). RNA (400 ng) was reverse transcribed using the High Capacity cDNA Reverse Transcription Kit (Thermo Fisher Scientific). For qPCR, primers for glyceraldehyde-3-phosphate dehydrogenase (forward: 5′-ACGGCAAATTCAACGGCAC-3′; reverse: 5′-TAGTGGGGTCTCGCTCCTGG-3′) and Il1b (forward: 5′-AACCTGCTGGTGTGTGACGTTC-3′; reverse: 5′-CAGCACGAGGCTTTTTTGTTGT-3′) were designed and purchased from Integrated DNA Technologies (Leuven, Belgium), or predesigned TaqMan gene expression assays were used (Thermo Fisher Scientific) (Table 1). All qPCR analyses were performed on a QuantStudio 6 Flex Real-Time PCR System (Applied Biosystems, Thermo Fisher Scientific). Target mRNA expression was normalized for RNA loading using glyceraldehyde-3-phosphate dehydrogenase (Integrated DNA Technologies), and the mRNA concentration in each sample was calculated relative to the vehicle control group (PBS or AAV8-GFP + PBS) using the 2^−ΔΔ^^C_T_^ method of analysis.

**Table 1:** Predesigned TaqMan Gene Expression Assays

Gene	TaqMan assay identifier
*Cxcl1*	Mm04207460_m1
*Cxcl2*	Mm00436450_m1
*Gfap*	Mm01253033_m1
*Iba1*	Mm00520165_m1
*Il6*	Mm00446190_m1
*Spa*	Mm00499170_m1
*Spb*	Mm00455678_m1
*Spc*	Mm00488144_m1
*Spd*	Mm00486060_m1
*Tnfa*	Mm00443258_m1

### Enzyme-Linked Immunosorbent Assay

E18.5 brains were homogenized in lysis buffer (radioimmunoprecipitation assay buffer; Merck Millipore, Darmstadt, Germany) containing 1 Complete Protease Inhibitor Cocktail Tablet (Roche, Basel, Switzerland) using a Tissue Lyser II (Qiagen) at 30 Hz. The samples were incubated on ice for 5 minutes and then centrifuged at 10,000 × *g* for 10 minutes at 4°C. The lysates were stored at –80°C. Total protein was quantified by using the DC protein assay (Bio-Rad, Hercules, CA), according to the manufacturer's instructions. The V-PLEX Proinflammatory Panel 1 Mouse Kit (Meso Scale Diagnostics, Rockville, MD) was used to quantify inflammatory mediator secretion, according to the manufacturer's guidelines, and measured on a Meso Sector S 600 mm. For E18.5 lungs and gut, total protein quantification was performed using the Pierce BCA Protein Assay kit (Thermo Fisher Scientific), then the MILLIPLEX mouse cytokine/chemokine magnetic bead panel (Luminex Multiplex Assay; R&D Systems, Minneapolis, MN) was used according to the manufacturer's guidelines and measured on a Luminex instrument with xPONENT software (Diasorin, Saluggia, Italy).

### Histology and Immunohistochemistry

Histologic staining included terminal transferase-mediated dUTP nick end labeling (TUNEL) with Co/Ni enhancement (Roche) performed following manufacturer's instructions, cresyl-violet (Nissl) staining, and hematoxylin and eosin staining. For brain histology and immunohistochemistry, five sections per brain (400 μm apart) were rehydrated in distilled water and left to dry. Immunohistochemical staining was performed as previously described.^23^ Briefly, tissue sections were incubated overnight with the following primary antibodies: rabbit polyclonal anti–glial fibrillary acidic protein (GFAP; 1:6000; Dako; Agilent Technologies, Inc., Santa Clara, CA), rabbit polyclonal anti–allograft inflammatory factor 1 (IBA1; microglia; 1:2000; Wako Company, Tokyo, Japan), rabbit anti–myelin basic protein (MBP; 1:200; Abcam, Cambridge, UK), or mouse monoclonal anti-neuronal nuclei (NeuN; 1:15,000; Merck Millipore). The following day, sections were incubated with either biotinylated goat anti-rabbit or anti-rat (1:100; Vector Laboratories, Newark, CA) secondary antibodies for 1 hour, then incubated with Avidin-Biotinylated horseradish peroxidase complex (Vector Laboratories), followed by diaminobenzidine/hydrogen peroxide (Thermo Fisher Scientific).

For lung analyses, two or three sections of lung per fetus or neonate (400 μm apart) were rehydrated in distilled water, then stained with Nissl, or immunohistochemical labeling was performed as above with anti-mouse lymphocyte antigen 6 complex locus G6D (Ly6G) primary antibody (BioLegend, London, UK) and goat anti-rabbit secondary antibody.

### Histologic Assessments

Assessments were performed on the following regions: cortex, pyriform cortex, external capsule, striatum, hippocampus, and thalamus. The corpus callosum was analyzed for E18.5 brains in addition to or in place of the external capsule.

#### Optical Luminosity

Optical luminosity values were generated to measure reactive astrogliosis and myelination through GFAP and myelin basic protein immunoreactivity, respectively. All regions were assessed for GFAP, and the striatum and external capsule were assessed for MBP. Images were captured in three optical fields using a Sony (Tokyo, Japan) AVT-Horn 184 3CCD camera [×20 magnification; 24-bit red, green, and blue (RGB); 760 × 570-pixel resolution], then imported into Optimas 6.51 (Media Cybernetics Inc., Rockville, MD) to determine the mean and SD of the optical luminosity value. To obtain the intensity of the staining for each image, the SD was subtracted from the mean optical luminosity value, and the resulting values were normalized by subtracting the optical luminosity value of the glass surrounding the tissue section.^24^

#### Ramification Index

IBA1-positive microglial cell bodies and branch density were assessed using a 0.049 × 0.049-mm square grid (×40 magnification) placed in three fields for all brain regions. The number of cell bodies within the grid were counted (C), and the average number of branches crossing the three horizontal and three vertical 0.49-mm gridlines was calculated (B). The microglial ramification index was calculated as follow: B^2^/C.

#### Stereology for Brain and Lung Tissues

TUNEL- (×20 magnification), NeuN- (×40 magnification), and Ly6G-positive cells (×40 magnification) were manually counted using ImageJ software version 1.53 (NIH, Bethesda, MD; *https://imagej.net*). Brain width, cortex thickness, hippocampal area, and lung airspace area were measured using the free-hand tool (×40 magnification). Three optical fields were assessed per brain region/lung tissue section, and an average was found.

### Statistical Analysis

Data were analyzed by using GraphPad Prism version 8 (GraphPad Software, La Jolla, CA). Time to delivery was analyzed by an unpaired *t*-test with Welsh correction or one-way analysis of variance with Sidak multiple comparisons test. The percentage data for live-born pups were analyzed by performing an arcsine transformation on the proportions, to normalize the binomial distribution, followed by an unpaired *t*-test with Welsh correction or one-way analysis of variance with Sidak multiple comparisons test. Pup survival was analyzed by the log-rank (Mantel-Cox) test. qPCR data were analyzed using an unpaired *t*-test with Welsh correction or one-way analysis of variance with Tukey multiple comparisons test. All enzyme-linked immunosorbent assay data were log transformed, followed by unpaired *t*-tests with Welsh correction. For the NF-κB biosensor data, the area under the curve was calculated, followed by an unpaired *t*-test. For histology and immunohistochemistry, either a *U*-test or multiple *t*-tests with Welsh correction were used. Scoring for histology and immunohistochemistry was blinded where possible. Experimental numbers were based on a sample size calculation that estimated that a minimum of five pups were needed to determine statistically significant differences between groups. Fetal and neonatal tissues were analyzed from a minimum of five independent litters per treatment group. *P* < 0.05 was considered statistically significant.

## Results

### Ascending Vaginal Infection with *E. coli* Reduces Gestational Length, the Percentage of Pups Born Alive, and Pup Survival over 7 Days

**Figure 1:**
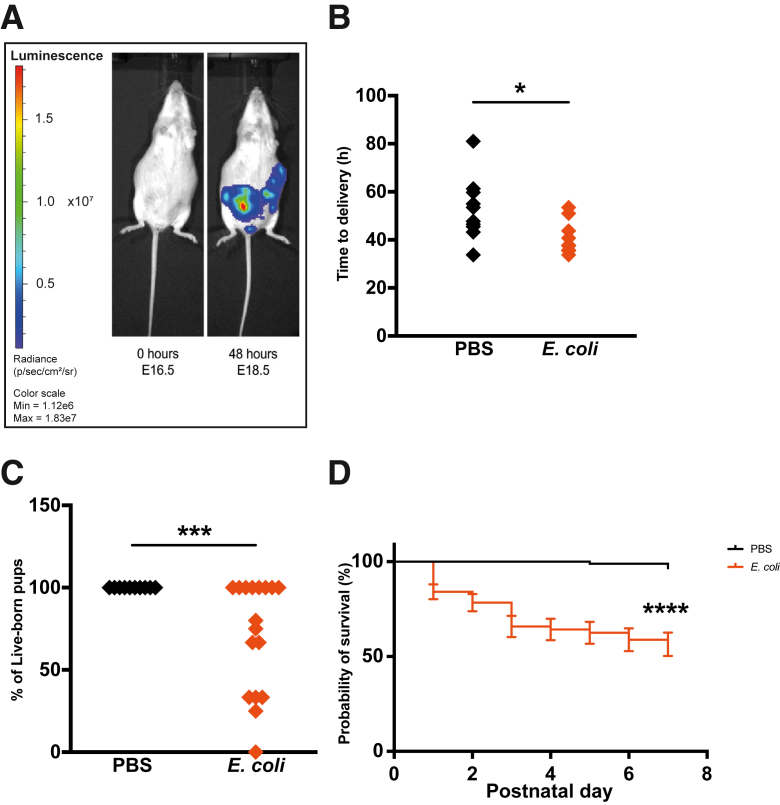
*Escherichia coli* ascends from the vagina into uterus to induce preterm birth and reduce pup survival. **A:** After 48 hours, bioluminescent *E. coli* was visible in the uterus. **B**–**D:***E. coli* reduced the time to delivery (**B**), the percentage of live-born pups (**C**), and the long-term survival of pups (**D**). *n* = 8 to 10 litters per group (**B**); *n* = 10 to 17 litters per group (**C**); *n* = 10 to 12 litters per group (**D**). ∗*P* < 0.05, ∗∗∗*P* < 0.001, and ∗∗∗∗*P* < 0.0001. E, embryonic day; Max, maximum; Min, minimum; PBS, phosphate-buffered saline.

On E16.5, 1 × 10^2^ colony-forming units of bioluminescent *E. coli* or 20 μL PBS control was administered to dams intravaginally. Imaging showed the ascension of the bacteria from the vagina into the uterus within 24 to 48 hours, (Figure 1A). Gestational length was significantly reduced in *E. coli*–treated dams compared with PBS-treated dams (Figure 1B) (mean time to delivery, 40.3 ± 7 versus 52.7 ± 12.9 hours for *E. coli* and PBS groups, respectively; *P* = 0.018). The average percentage of pups born alive was reduced by almost 30% following *E. coli* exposure (71.4% ± 34% versus 100% ± 0% in the PBS group; *P* = 0.001; Figure 1C), and postnatal survival over 7 days was also reduced (*P* < 0.0001; Figure 1D).

### Exposure to Ascending Vaginal Infection *in Utero* Induces Perinatal Neuroinflammation

**Figure 2:**
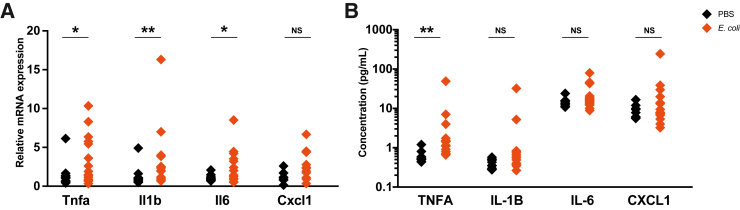
Ascending *Escherichia coli* infection leads to perinatal neuroinflammation 48 hours after exposure. Proinflammatory mediators tumor necrosis factor alpha (Tnfa), Il1b, and Il6 were up-regulated at the mRNA level (**A**), and TNFA was up-regulated at the protein level (**B**), in the perinatal pup brain following maternal *E. coli* infection. Tnfa, Il1b, and Il6: *n* = 15 from 5 litters per group; cxcl1: *n* = 8 to 13 from 5 litters per group (**A**); protein: phosphate-buffered saline (PBS) *n* = 10 from 5 litters; *E. coli n* = 15 from 5 litters (**B**). ∗*P* < 0.05, ∗∗*P* < 0.01. NS, nonsignificant.

On E18.5, 48 hours after maternal vaginal infection with *E. coli*, perinatal pup tissues were collected and assessed for inflammation and pathology. Inflammatory mediator mRNA expression was up-regulated with *E. coli* exposure (Figure 2A): tumor necrosis factor alpha (Tnfa) (*P* = 0.025), Il1b (*P* = 0.01), and Il6 (*P* = 0.034). TNFA secretion was also significantly elevated (Figure 2B) (0.6 ± 0.2 versus 4.9 ± 12.3 pg/mL in PBS and *E. coli* pups, respectively; *P* = 0.003). The secretion of IL-1B, IL-2, IL-4, IL-5, IL-6, IL-10, CXCL1, and interferon gamma (IFNG) was unchanged (Figure 2B and Supplemental Figure S1A).

Histologic analyses suggested a trend toward TUNEL^+^ cell death, particularly in the corpus callosum and striatum (Supplemental Figure S1B), and trends for reduced microglial ramification were seen in the cortex and thalamus (Supplemental Figure S1C). The average numbers of NeuN^+^ neurons in the cortex and hippocampus were not altered by *E. coli* exposure at this time point, and morphologic changes were not observed (Supplemental Figure S1, D–G).

### *E. coli* Exposure *in Utero* Leads to Neonatal Neuropathology

**Figure 3:**
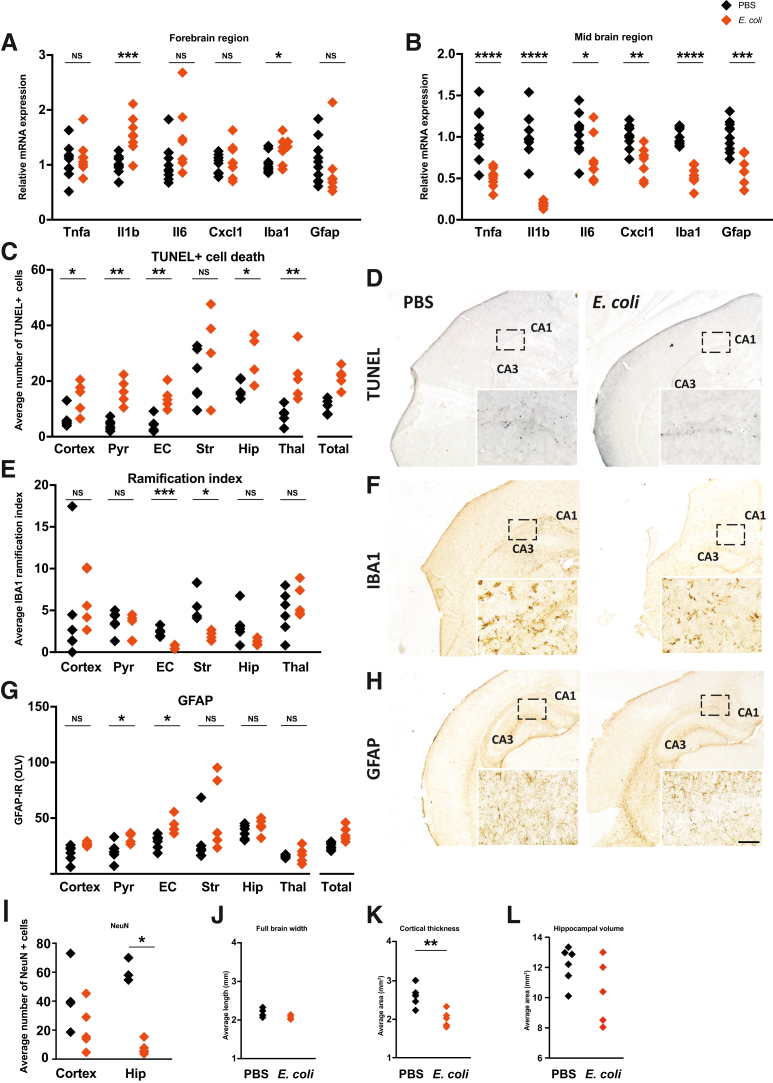
Neuropathology is evident in neonates (postnatal day 7) exposed to *Escherichia coli in utero*. **A** and **B:** Inflammatory mediators Il1b and allograft inflammatory factor 1 (Iba1) were up-regulated in the forebrain (**A**), whereas all inflammatory mediators were down-regulated in the mid brain region (**B**). **C** and **D:** The average number of terminal transferase-mediated dUTP nick end labeling (TUNEL)^+^ cells was increased in most brain regions (**C**), and representative images are shown (**D**). **E** and **F:** IBA1^+^ cell ramification was reduced in the external capsule (EC) and striatum (Str) of the brain in the *E. coli* group (**E**), and representative images are shown (**F**). **G** and **H:** Glial fibrillary acidic protein (GFAP) was increased in the pyriform cortex (Pyr) and the external capsule (**G**), and representative images are shown (**H**). **I:** The average number of neuronal nuclei (NeuN)^+^ cells was reduced in the hippocampus (Hip). **J**–**L:** There was no impact on brain width (**J**) or hippocampal volume (**L**), but the cortical thickness was reduced in the *E. coli* group (**K**). **Dashed line boxed areas** denote the regions where the insert image was captured. mRNA: *n* = 8 to 10 from 5 litters (**A** and **B**); immunohistochemistry analyses: *n* = 5 to 7 from 5 litters per group (**C**–**L**). ∗*P* < 0.05, ∗∗*P* < 0.01, ∗∗∗*P* < 0.001, and ∗∗∗∗*P* < 0.0001. Scale bar = 59.5 μm (**D**, **F**, and **H**). CA, cornu ammonis; IR, immunoreactivity; NS, nonsignificant; OLV, optical luminosity value; PBS, phosphate-buffered saline; Thal, thalamus; Tnfa, tumor necrosis factor alpha; Total, all regions.

At P7, Il1b and Iba1 mRNA, a marker of microglia, were up-regulated in the forebrain region (*P* = 0.001 and *P* = 0.016, respectively) (Figure 3A). In the mid brain region, mRNA expression was down-regulated (Figure 3B): Tnfa (*P* < 0.0001), Il1b (*P* < 0.0001), Il6 (*P* = 0.028), Cxcl1 (*P* = 0.005), Iba1 (*P* < 0.0001), and Gfap (*P* = 0.0006). Luciferase expression, signifying NF-κB brain signaling, was also reduced in the *E. coli* neonatal brains (*P* = 0.002) (Supplemental Figure S2, A and B).

Histologic analyses found widespread TUNEL^+^ cell death in *E. coli* pup brains: cortex (*P* = 0.013), pyriform cortex (*P* = 0.004), external capsule (*P* = 0.004), hippocampus (*P* = 0.019), and thalamus (*P* = 0.004) (Figure 3, C and D). There was reduced microglial ramification (Figure 3, E and F) in the external capsule (*P* = 0.001) and striatum (*P* = 0.011). GFAP, a marker of astrogliosis, was significantly increased in the pyriform cortex (Figure 3, G and H) (*P* = 0.027) and external capsule (*P* = 0.027). *E. coli*–exposed pups also had reduced numbers of NeuN^+^ neurons in the hippocampal region of the brain (Figure 3I) (*P* = 0.036), and the cortical area was reduced in these pups (Figure 3K) (*P* = 0.009). There was no difference in fetal brain width (Figure 3J) or in hippocampal volume (Figure 3L).

**Figure 4:**
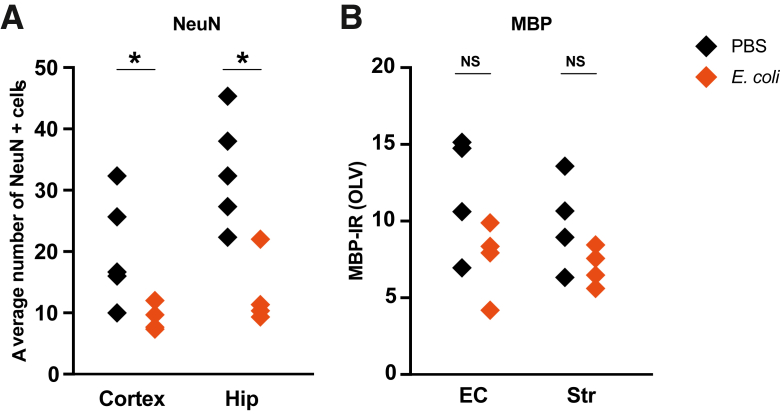
Neuronal density is reduced at postnatal day 14 following *in utero Escherichia coli* exposure. **A:** The average number of neuronal nuclei (NeuN)^+^ cells was reduced in the cortex and hippocampus (Hip). **B:** There was no significant change to myelin basic protein (MBP) luminosity. *n* = 4 to 5 per group from ≥4 litters (**A** and **B**). ∗*P* < 0.05. EC, external capsule; IR, immunoreactivity; NS, nonsignificant; OLV, optical luminosity value; PBS, phosphate-buffered saline; Str, striatum.

At P14, neuronal density was reduced in the cortex (Figure 4A) (*P* = 0.032) and the hippocampus (*P* = 0.016). There was no longer evidence of TUNEL^+^ cell death or astrogliosis, and MBP staining intensity was not significantly altered (Figure 4B and Supplemental Figure S2, C and D).

### Exposure to Ascending Vaginal Infection *in Utero* Induces Inflammation in the Perinatal Lungs and Gut

#### Lung

**Figure 5:**
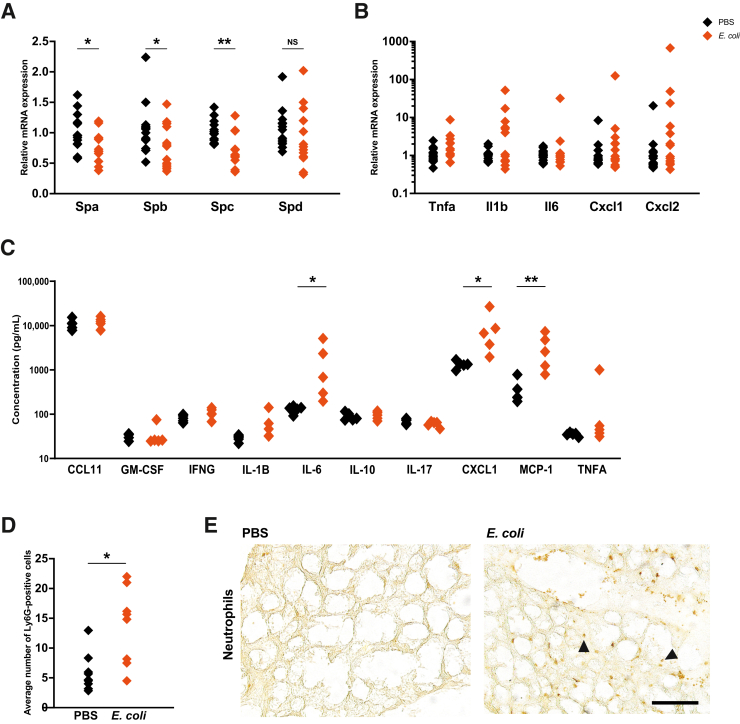
There is evidence of injury to the perinatal lungs following *Escherichia coli* exposure. **A:** Surfactant protein (Sp) mRNA expression was reduced in the *E. coli* group of embryonic day 18.5 pups. **B** and **C:** A trend for increased inflammatory mediator expression could be seen at the mRNA level (**B**), and significant increases were observed at the protein level (**C**). **D** and **E:** The average number of Ly6G^+^ cells (**black arrowheads**) was increased. *n* = 12 to 15 from 5 litters per group (**B**); *n* = 5 from 5 litters per group (**C**); immunohistochemistry: *n* = 10 from 5 litters per group (**D** and **E**). ∗*P* < 0.05, ∗∗*P* < 0.01. Scale bar = 59.5 μm (**E**). CCL11, chemokine (C-C motif) ligand 11; GM-CSF, granulocyte-macrophage colony-stimulating factor; IFNG, interferon gamma; MCP-1, monocyte chemoattractant protein 1; NS, nonsignificant; PBS, phosphate-buffered saline; TNFA, tumor necrosis factor alpha.

Surfactant protein (Sp) mRNA expression was down-regulated in the lungs of pups from *E. coli*–treated dams (Figure 5A): Spa (*P* = 0.023), Spb (*P* = 0.034), and Spc (*P* = 0.006). There was also pup-dependent elevation of inflammatory mediator mRNA expression but, overall, there was no significant change to any of these markers compared with that in the PBS group (Figure 5B). However, IL-6 (*P* = 0.035), CXCL1 (*P* = 0.02), and monocyte chemoattractant protein 1 (*P* = 0.004) were increased in these mice (Figure 5C). Additionally, there was a significant increase in Ly6G-positive neutrophil infiltration in the lungs of *E. coli*–exposed pups (Figure 5, D and E) (*P* = 0.011), and the mean airspace area was increased (Supplemental Figure S3, A and B) (*P* = 0.0112). Neutrophil influx and morphologic changes were no longer evident at P7 (Supplemental Figure S3, C and D).

#### Gut

**Figure 6:**
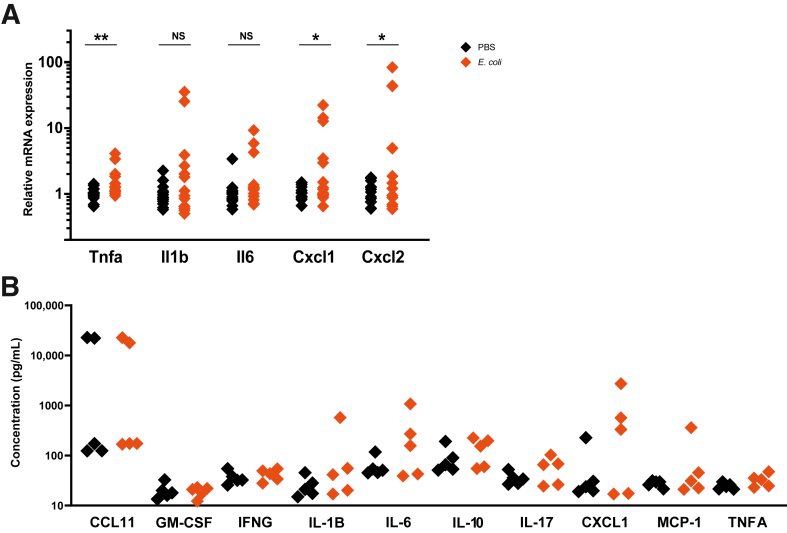
*Escherichia coli* induces perinatal gut inflammation. **A:** Inflammatory mediator mRNA expression was increased in the gut. **B:** There was no change to the protein expression of these mediators. *n* = 14 to 15 from 5 litters per group (**A**); *n* = 5 from 5 litters per group (**B**). ∗*P* < 0.05, ∗∗*P* < 0.01. CCL11, chemokine (C-C motif) ligand 11; GM-CSF, granulocyte-macrophage colony-stimulating factor; IFNG, interferon gamma; MCP-1, monocyte chemoattractant protein 1; NS, nonsignificant; PBS, phosphate-buffered saline; TNFA, tumor necrosis factor alpha.

In the gut, Tnfa (*P* = 0.007), Cxcl1 (*P* = 0.031), and Cxcl2 (*P* = 0.038) mRNA expression levels were elevated, but there was no impact on Il1b, Il6, or Il10 mRNA expression or on the secretion of inflammatory proteins (Figure 6, A and B).

### Maternal AAV8-HBD3 Cervical Gene Therapy Improves Pup Survival but Does Not Increase Gestational Length

**Figure 7:**
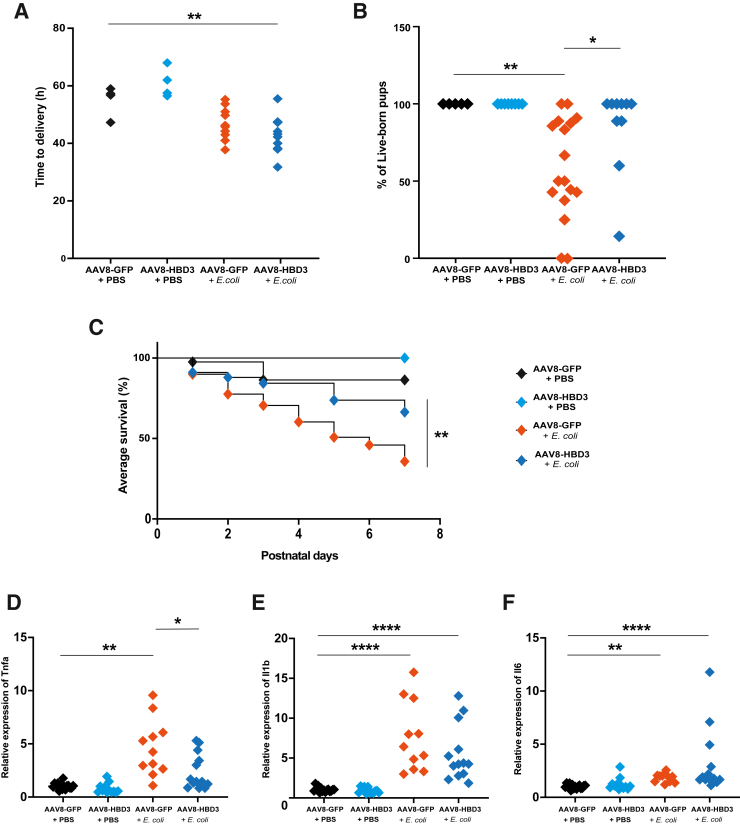
Cervical human β defensin 3 (HBD3) gene delivery improves pup survival and reduces tumor necrosis factor alpha (Tnfa) in the perinatal brain. **A**–**C:** HBD3 treatment did not impact the length of gestation (**A**), but increased the percentage of live-born pups (**B**) and long-term pup survival (**C**). **D**–**F:** Tnfa, but not Il1b and Il6, was reduced in the perinatal brain following maternal cervical HBD3 gene delivery. Delivery and survival: adeno-associated virus 8 (AAV8)–green fluorescent protein (GFP) + phosphate-buffered saline (PBS) *n* = 5 litters, AAV8-HBD3 + PBS *n* = 8 litters, AAV8-GFP + *Escherichiacoli n* = 17 litters, AAV8-HBD3 + *E. coli n* = 10 litters (**A**–**C**); mRNA: *n* = 11 to 15 from 5 litters per group (**D**–**F**). ∗*P* < 0.05, ∗∗*P* < 0.01, and ∗∗∗∗*P* < 0.0001.

To validate the utility of the model, AAV8-HBD3 was administered. First, the effect on PTB was evaluated. The gestational length of infected dams was not increased with maternal AAV8-HBD3 treatment compared with the AAV8-GFP + *E. coli* group (42.54 ± 6 versus 46.55 ± 5 hours, respectively) (Figure 7A). The percentage of live-born pups was significantly increased in comparison to the AAV8-GFP + *E. coli* group (Figure 7B) (85.2% versus 58.6%, respectively; *P* = 0.043), and pup survival was improved over 7 days (Figure 7C) (*P* = 0.002). This is consistent with previous data using this treatment.^19^

This model was also used to examine the therapeutic effect on perinatal brain pathology. AAV8-HBD3 reduced Tnfa mRNA expression in the E18.5 pup brains exposed to *E. coli* compared with control vector (*P* = 0.008; Figure 7D) but did not significantly alter Il1b or Il6 mRNA expression (Figure 7, E and F).

## Discussion

PTB, and the resulting neonatal morbidity and mortality, is a serious global health issue for which preventative treatments are largely ineffective. It is essential to establish clinically relevant models to recapitulate not only preterm delivery but also the impact this has on the fetus to develop innovative interventions. It is possible to replicate ascending vaginal infection in mice.^18^ Here, neuropathology, lung injury, and gut inflammation, key neonatal morbidities associated with prematurity, were modeled. Furthermore, this model was used to investigate cervical gene delivery of HBD3, revealing that this intervention can significantly increase pup survival while reducing Tnfa expression in the perinatal brain.

### Modeling Ascending Vaginal Infection and PTB

This mouse model is the only one, to our knowledge, that recapitulates the human condition, whereby PTB is induced by live bacterial ascending infection, that also reduces pup survival and causes neonatal morbidity in the survivors. Mice are commonly used to model human PTB. There are several methods used to induce labor, but by far the most common method is via lipopolysaccharide (LPS) administration.^25^ However, there is huge heterogeneity among models, which differ in the mouse strain used, LPS serotype, LPS dose, mode of delivery, and day of induction.^26^ LPS models have several disadvantages. The use of a bacterial product rather than live bacteria means it is a model of inflammation, not infection, and the clinical scenario of ascension through the cervix cannot be recapitulated because of LPS immotility. In addition, the dose required to induce PTB is often lethal for fetuses, preventing neonatal morbidity studies.^27^

Recently, another study characterized a similar model of *E. coli*–induced PTB, demonstrating bacterial ascension, maternal inflammation, and early delivery, validating the current results. However, regardless of *E. coli* dose, there were no surviving pups.^28^^,^^29^ In a later study, this group reported 47.1% live-born pups when combining the vaginal delivery of a low dose of *Ureaplasma parvum* and *E. coli*. However, neonatal outcomes were not assessed.^30^ Others have modeled ascending infection with bacteria of particular relevance to human PTB, such as *U. parvum* and Group B *Streptococcus*.^31^, ^32^, ^33^, ^34^, ^35^ However, these bacteria do not consistently induce labor in mice, and the impact on pups has not been investigated beyond recording survival immediately after birth. Although *E. coli* is not a common organism implicated in PTB, it is associated with preterm sequelae in mothers and is often associated with poor neonatal outcomes.^36^, ^37^, ^38^ Specifically, *E. coli* K1 strains are commonly acquired from the mother at birth and are associated with neonatal sepsis and meningitis.^39^ Indeed, the dams can often become unwell with this pathogenic strain. However, they usually recover following parturition.

### Neuropathology

Markers of neuroinflammation were evident during the perinatal period at both the mRNA and protein level in pups exposed to *E. coli*, which is in agreement with mouse models of intrauterine inflammation induced by LPS.^40^, ^41^, ^42^, ^43^, ^44^, ^45^, ^46^ The developing brain is extremely sensitive to inflammation; an adverse environment even in an asymptomatic mother could cause brain injury in the fetus, and cytokines are known to be neurotoxic.^47^ Maternal infection and inflammation may alter the permeability of the fetal blood-brain barrier, leading to neuroinflammation. Through exposure to excess inflammatory mediators, immune cells can accumulate at the embryonic choroid plexus, weakening tight junctions at this barrier.^48^ It is hypothesised that the neuroinflammation is due to systemic inflammation and potentially infection in the pups. Evidence of *E. coli* in the gastrointestinal and respiratory tracts has been previously published.^18^ Although pups were not assessed for infection in this current study, as the *E. coli* K1 strain is associated with meningitis, it is possible that the microbes could cross the blood-brain barrier.

While there was a visible trend for reduced ramification of microglia at E18.5, suggesting phagocytic activity, neuropathology was not fully evident until P7, which is likely the result of this perinatal neuroinflammation. A limitation of this model is the variation seen both within and between litters, where some pups exhibit significantly more inflammatory mediator expression than others. As live bacteria were administered, it was impossible to control the spread of the infection, but the position of fetuses in the uterine horns may also have played a role. In future studies, further exploration is needed to understand the patterns of ascending infection into the uterine horns. It would also be worthwhile to increase the group numbers, so that the true impact of infection can be appreciated.

Only minimal inflammation, restricted to the prefrontal cortex region of the brain, remained by P7, with modest increases in Il1b and the microglial marker, Iba1. Interestingly, mRNA expression of all mediators assessed was reduced in the mid brain region (consisting mainly of the hippocampus, thalamus, and striatum) of these mice. This lack of inflammatory mRNA expression could be the result of substantial apoptosis. There were activated microglia in the hippocampus, evidenced by a phagocytic phenotype, which may be associated with the increased TUNEL^+^ cell death and reduced neuronal density in this region. Alternatively, both this down-regulation in inflammatory mediators and neuronal density could be explained by the reduction in NF-κB activity. NF-κB is a key modulator of inflammatory genes, but low levels of NF-κB response in the brain are also associated with neuronal apoptosis.^49^ In future studies, dissection of individual brain regions would provide a fuller picture of the mRNA expression changes at this time point.

Astrogliosis can occur in response to neuronal loss following central nervous system injury. These cells release cytokines that may exacerbate inflammation and injury, but they also restore homeostasis and promote neurogenesis.^50^ However, GFAP staining was not increased in the hippocampus at P7, which could suggest that neurogenesis was not stimulated by astrocytes in this region, resulting in the consistently reduced neuronal density at P7 and P14.

In addition to reduced neurogenesis, activation of both microglia and astroglia was increased across multiple brain regions, which has been reported in intrauterine inflammation models.^51^, ^52^, ^53^ TUNEL^+^ cell death was evident throughout the brain. White matter injury, periventricular leukomalacia, and cerebral palsy are associated with apoptosis and necrosis in the brain.^54^^,^^55^ Chorioamnionitis-associated white matter injury is common in preterm infants and is thought to begin *in utero*.^56^ It is associated with neuronal cell death, microglial activation, and disruption to oligodendrogenesis, which can result in a myelination deficit.^56^^,^^57^ In the current model, microglial activation was increased, as was cell death and astrogliosis, in the external capsule at P7, suggesting white matter injury pathognomonic of PTB brain injury.

By P14, measurable cell death or astrogliosis was no longer observed, but neuronal density was still reduced in the hippocampus and the cortex. This suggests that although neuroinflammation may have subsided, there was a lasting effect on neurogenesis. There did not appear to be disruption to myelination at P14; however, there is no report of this change until P30 in the LPS mouse model.^44^

Cortical area was reduced in *E. coli*–exposed pups, similarly to prematurely born humans, where reduced cortical thickness has been described.^58^, ^59^, ^60^ Neuroimaging has confirmed reduced white and gray matter volumes in preterm infants, and the microstructure of the cerebral cortex is altered in regions associated with cognition and sensory and motor function.^61^^,^^62^ Hippocampal volume was not reduced, contrary to what has been reported from other animal models.^52^^,^^63^

Here, exposure to ascending vaginal infection during gestation caused disrupted neurodevelopment, which in humans can lead to cognitive and behavioral issues, with an increased risk of cerebral palsy, attention-deficit/hyperactivity disorder, autism spectrum disorder, and psychiatric issues.^64^

### Lung Injury

Fetal lungs are susceptible to injury when exposed to infection and/or inflammation via the amniotic fluid.^65^ This results in an influx of neutrophils and increased inflammation and can impact alveolarization and surfactant production.^66^ Interestingly, inflammatory mediator mRNA expression was only up-regulated in specific pups in our model. This could be a result of the positioning of the pups within the uterine horns. Nevertheless, certain secreted inflammatory mediators were elevated, and the average number of neutrophils in the lungs was increased, which suggests that a measurable level of inflammation was occurring. Indeed, other models have reported an increase in inflammatory mediators^67^, ^68^, ^69^ and neutrophil influx into the fetal lungs.^69^^,^^70^

Pulmonary surfactants, important for reducing surface tension, are essential for respiration.^71^ Surfactant protein mRNA expression was reduced in the perinatal pup lungs, which is supported by data from other murine models.^67^^,^^72^ Surfactant deficiency and lung morphologic disruption can result in respiratory distress syndrome and consequent ventilatory support, which can subsequently increase susceptibility to conditions such as bronchopulmonary dysplasia and chronic lung disease of prematurity.^73^ The morphology of the fetal lungs was also disrupted, evidenced by increased airspace area. This has been reported in mouse, sheep, and nonhuman primate models, and may be the result of activated immune cells in the lung.^68^^,^^74^, ^75^, ^76^

A limitation of using mice to model prenatal lung injury is that alveologenesis occurs postnatally in mice, beginning around P4 and peaking at P7, rather than before term, as in humans and nonhuman primates.^65^^,^^77^ This could explain why lung maturation does not appear to be accelerated in this model. An additional limitation was the collection and processing of these tissues, as the lungs were not inflated. Nevertheless, our model does appear to mimic the morphologic changes seen in neonatal bronchopulmonary dysplasia models.^78^^,^^79^

### Gastrointestinal Inflammation

There was a marked increase in inflammatory cytokine mRNA expression in the perinatal gut. This particular strain of *E. coli* is known to colonize the gastrointestinal tract, which has been demonstrated in neonatal rats. The bacteria cross the epithelium of the gut into the systemic circulation and enter the central nervous system by crossing the blood-brain barrier, via choroid plexus epithelium, where they invade the meninges.^39^^,^^80^^,^^81^ One study demonstrated increased gut inflammation and increased gut permeability in naturally preterm pups, showing that, even in the absence of infection, prematurity alone is enough to disrupt the gut.^82^

A neonatal mouse model of NEC describes disruption to the intestinal mucosa and increased Il1b and Cxcl2 expression.^83^ As the immune system of premature babies may be underdeveloped or dysregulated, they are susceptible to conditions such as NEC, resulting from an exacerbated inflammatory response to microbial products introduced during feeding.^84^ In addition, premature infants have delayed bacterial colonization and reduced microbial diversity in the gut. This can have an effect for up to 4 years following disruption during the perinatal period.^85^, ^86^, ^87^ NEC, itself, can result in brain damage because of increased permeability of the gut caused by damage to the intestinal epithelium, leading to bacterial translocation into the blood. The activation of T cells or neurotoxic levels of IFN-γ can activate microglia and lead to disrupted myelination.^88^ Although NEC is believed to be established postnatally, we may be modelling a similar pathology. In addition, the colonization of the gut postnatally may contribute to the neonatal neuropathology observed.

### Validating the Model by Cervical HBD3 Gene Therapy

Advanced therapies like gene delivery may offer an innovative solution for those at risk of preterm delivery, which has proved difficult to prevent and treat. Although gene therapy is still in the relatively early stages of clinical testing and clinical use, there are increasing numbers of trials investigating gene and cell delivery during pregnancy.^89^, ^90^, ^91^, ^92^ It was hypothesized that facilitating the up-regulation of the antibacterial peptide HBD3 would enhance the immunologic barrier of the cervix, thus protecting the developing fetus from morbidity and mortality. The current model of ascending vaginal infection was used to validate the previous finding that this gene therapy increases the number of live-born pups.^19^ These results have been expanded upon, confirming that postnatal pup survival was also improved, and there was a reduction in Tnfa mRNA expression in the perinatal pup brain, suggesting that this treatment may shield pups from the damaging levels of inflammation resulting from maternal infection. Human neonatal brain damage is associated with elevated TNFA in cord blood.^93^, ^94^, ^95^ Activated microglia release TNFA, but these cells can also be activated by TNFA via a positive feedback loop, resulting in sustained activation.^96^ Therefore, by reducing Tnfa, this treatment may prevent neuropathology mediated by cytokine toxicity and microgliosis. Although HBD3 gene therapy did not prevent PTB in this model, it played an arguably more important role in improving pup survival and reducing inflammation in the perinatal brain. Therefore, this treatment strategy warrants further investigation, including a full analysis of the neonatal brain to determine whether this therapy protects pups from neuroinflammation and brain cell death in the longer-term.

## Conclusion

It is essential to develop clinically relevant animal models, such as the one described here, to investigate the underlying mechanisms leading to PTB and for the advancement of novel therapies. This mouse model of ascending vaginal infection induced PTB and reduced pup survival but, importantly, this model also recapitulated neonatal morbidity. This includes lung injury, gut inflammation, and neonatal neuropathology, all of which are fundamental to the conditions premature babies are susceptible to. This model was also used to investigate novel therapeutics targeting PTB and the associated neonatal morbidities.

## Disclosure Statement

None declared.

## Supplemental Data

Supplemental Figure S1 Perinatal neuropathology assessments. **A:***Escherichia coli* exposure did not impact the protein expression of IL-2, IL-4, IL-5, IL-10, or interferon gamma (IFNG) in perinatal brains. **B**–**G**: There was no significant difference in the number of terminal transferase-mediated dUTP nick end labeling (TUNEL)^+^ cells (**B**), allograft inflammatory factor 1 (IBA1)^+^ microglial ramification (**C**), neuronal nuclei (NeuN)^+^ cells (**D**), or brain morphology (**E**–**G**). Protein: phosphate-buffered saline (PBS) *n* = 10 from 5 litters; *E. coli n* = 15 from 5 litters; TUNEL, NeuN, and Nissl: *n* = 4 to 5 pups from ≥4 litters per group (**A**, **B**, and **D**); IBA1: *n* = 2 to 5 (dependant on region) from individual litters per group (**C**). Original magnification, ×1 (**G**). CC, corpus callosum; EC, external capsule; Hip, hippocampus; Pyr, pyriform cortex; Str, striatum; Thal, thalamus.

Supplemental Figure S2 Neonatal neuropathology assessments. **A:** Intracranial luciferase expression, as a measurement of NF-κB signaling, was observed in neonatal brains up to postnatal day (P) 21. **B:** There was a reduction in NF-κB signaling following *Escherichia coli* exposure. **C** and **D:** P14 terminal transferase-mediated dUTP nick end labeling (TUNEL) cell death (**C**) and glial fibrillary acidic protein (GFAP) activity (**D**) were unaltered. *n* = 4 to 7 per group from ≥4 litters (**A**–**D**). ∗∗*P* < 0.01. EC, external capsule; Hip, hippocampus; IC, intracranial; IR, immunoreactivity; OLV, optical luminosity value; PBS, phosphate-buffered saline; Pyr, pyriform cortex; Str, striatum; Thal, thalamus; Total, all regions.

Supplemental Figure S3 Perinatal and neonatal lung assessments. **A** and **B:** The average airspace area was increased in fetuses exposed to *Escherichia coli* compared with phosphate-buffered saline (PBS). **C** and **D:** However, there were no differences in the lung morphology or neutrophil influx at postnatal day 7. Scale bar = 59.5 μm (**B**). Perinatal: *n* = 2 pups from ≥4 litters per group (**A** and **B**); neonatal: *n* = 3 to 4 pups per group from ≥3 litters (**C** and **D**). ∗*P* < 0.05.
