# Supplementary figures and images for: Ascending Vaginal Infection in Mice Induces Preterm Birth and Neonatal Morbidity
Source: Am J Pathol. 2025 Jan 30;195(5):891–906. doi: 10.1016/j.ajpath.2025.01.008 (PMC12179526; doi:10.1016/j.ajpath.2025.01.008)

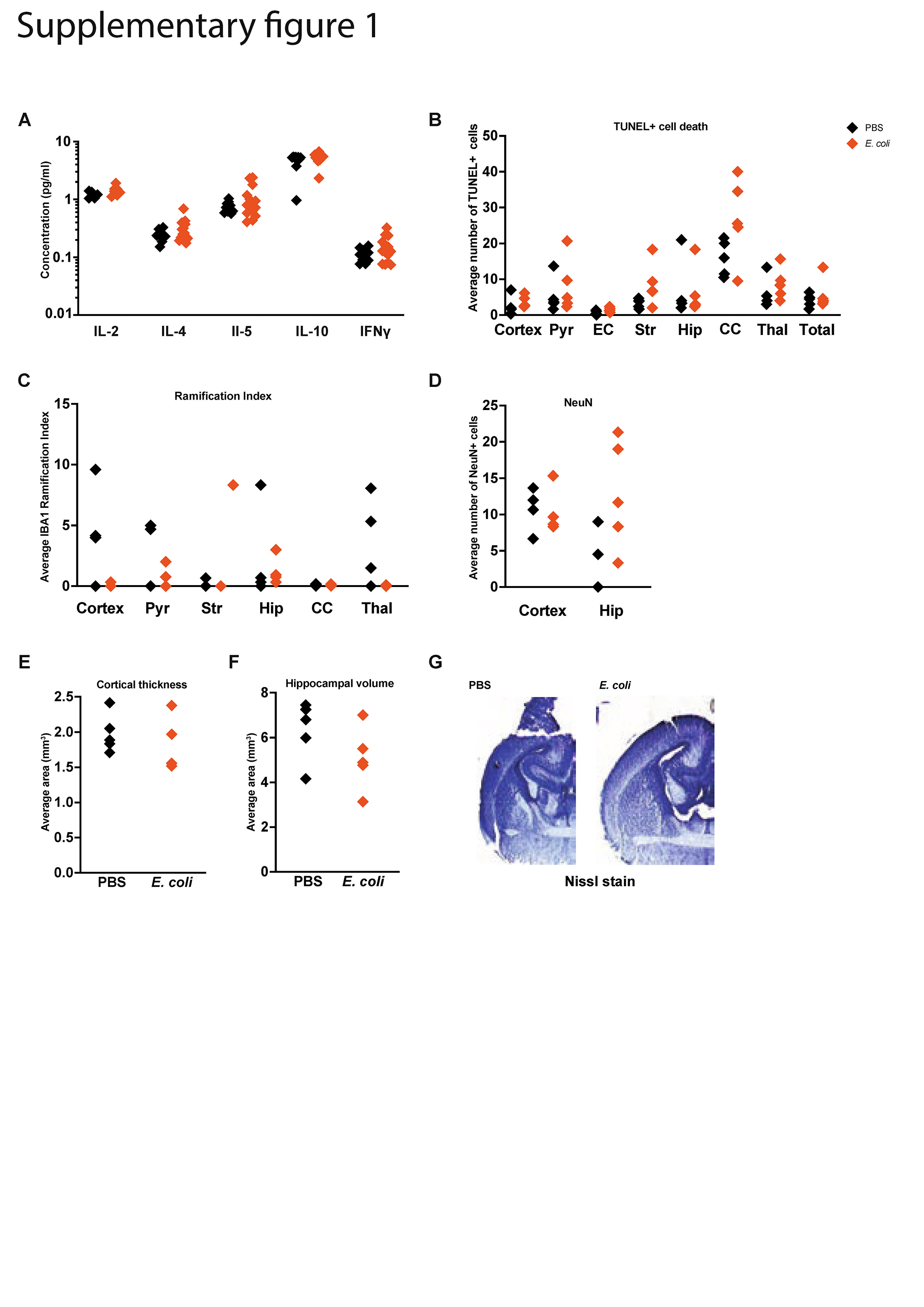

Supplement: Supplemental Figure S1 — Perinatal neuropathology assessments. A:Escherichia coli exposure did not impact the protein expression of IL-2, IL-4, IL-5, IL-10, or interferon gamma (IFNG) in perinatal brains. B–G: There was no significant difference in the number of terminal transferase-mediated dUTP nick end labeling (TUNEL)+ cells (B), allograft inflammatory factor 1 (IBA1)+ microglial ramification (C), neuronal nuclei (NeuN)+ cells (D), or brain morphology (E–G). Protein: phosphate-buffered saline (PBS) n = 10 from 5 litters; E. coli n = 15 from 5 litters; TUNEL, NeuN, and Nissl: n = 4 to 5 pups from ≥4 litters per group (A, B, and D); IBA1: n = 2 to 5 (dependant on region) from individual litters per group (C). Original magnification, ×1 (G). CC, corpus callosum; EC, external capsule; Hip, hippocampus; Pyr, pyriform cortex; Str, striatum; Thal, thalamus. [file figs1.jpg]

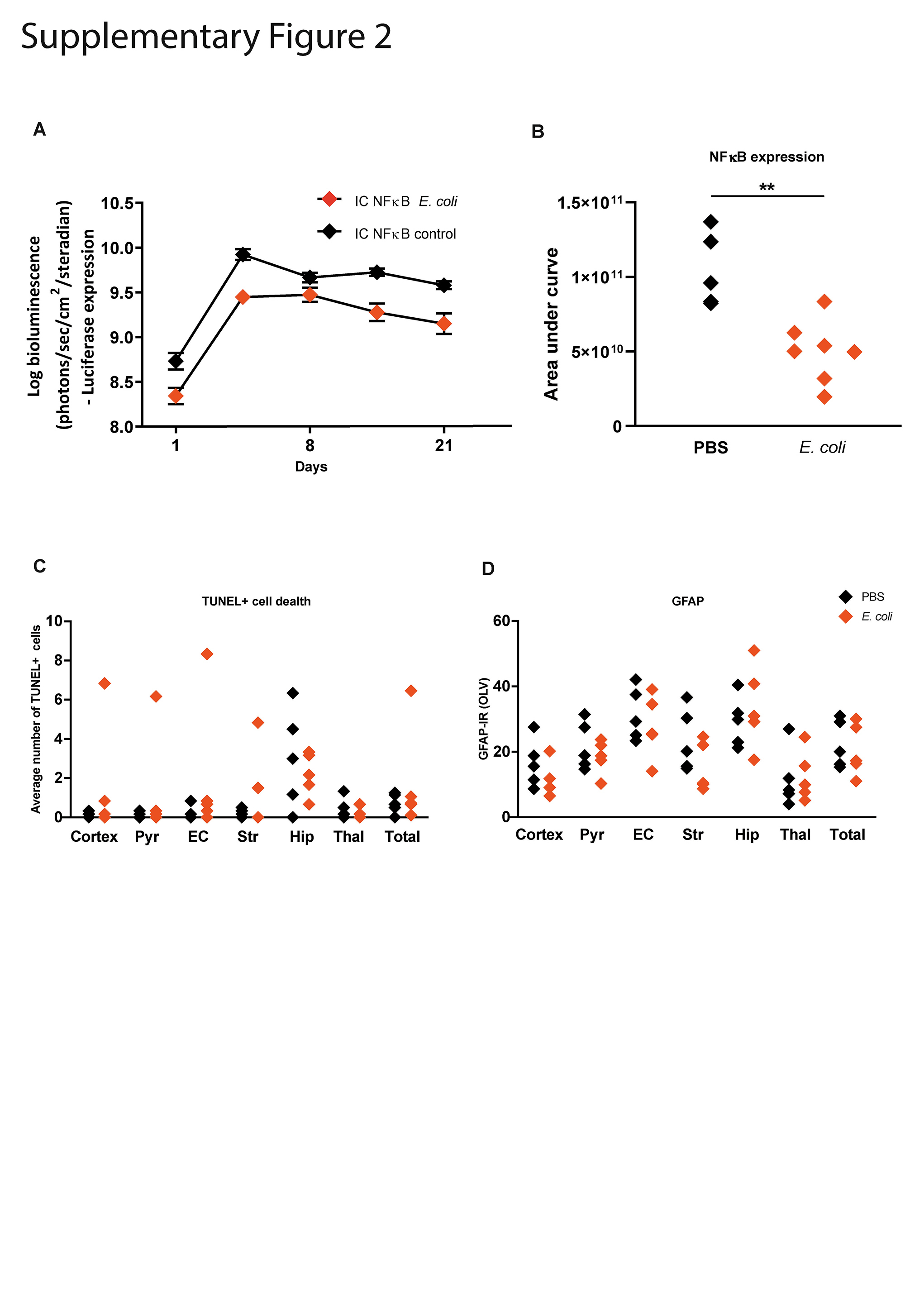

Supplement: Supplemental Figure S2 — Neonatal neuropathology assessments. A: Intracranial luciferase expression, as a measurement of NF-κB signaling, was observed in neonatal brains up to postnatal day (P) 21. B: There was a reduction in NF-κB signaling following Escherichia coli exposure. C and D: P14 terminal transferase-mediated dUTP nick end labeling (TUNEL) cell death (C) and glial fibrillary acidic protein (GFAP) activity (D) were unaltered. n = 4 to 7 per group from ≥4 litters (A–D). ∗∗P < 0.01. EC, external capsule; Hip, hippocampus; IC, intracranial; IR, immunoreactivity; OLV, optical luminosity value; PBS, phosphate-buffered saline; Pyr, pyriform cortex; Str, striatum; Thal, thalamus; Total, all regions. [file figs2.jpg]

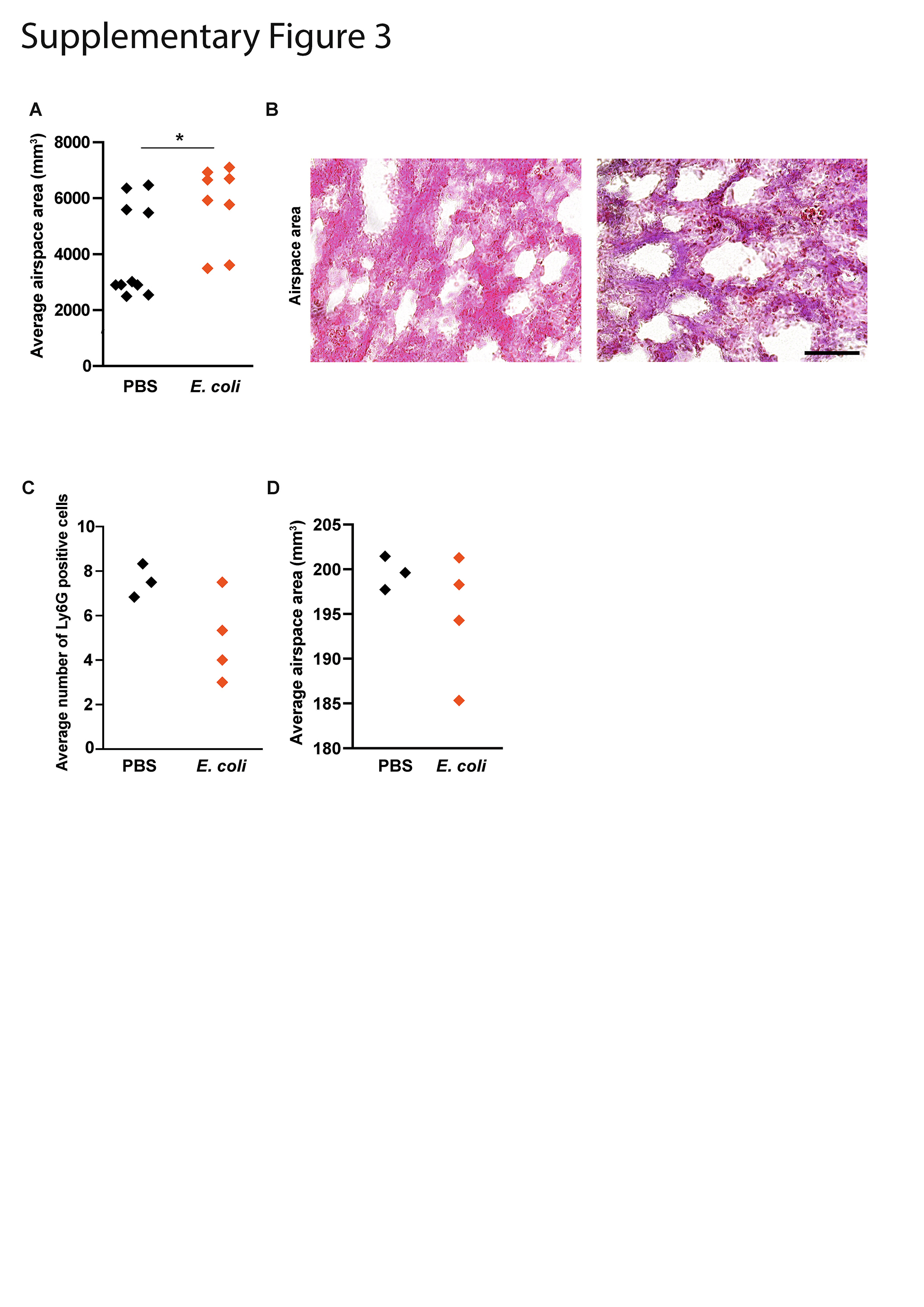

Supplement: Supplemental Figure S3 — Perinatal and neonatal lung assessments. A and B: The average airspace area was increased in fetuses exposed to Escherichia coli compared with phosphate-buffered saline (PBS). C and D: However, there were no differences in the lung morphology or neutrophil influx at postnatal day 7. Scale bar = 59.5 μm (B). Perinatal: n = 2 pups from ≥4 litters per group (A and B); neonatal: n = 3 to 4 pups per group from ≥3 litters (C and D). ∗P < 0.05. [file figs3.jpg]
